# Invasive Fungal Infections in Patients with Hematological Malignancies: Emergence of Resistant Pathogens and New Antifungal Therapies

**DOI:** 10.4274/tjh.2018.0007

**Published:** 2018-03-06

**Authors:** Maria N. Gamaletsou, Thomas J. Walsh, Nikolaos V. Sipsas

**Affiliations:** 1 The Leeds Teaching Hospitals NHS Trust, St James University Hospital, Department of Infection and Travel Medicine, Leeds, United Kingdom; 2 Weill Cornell Medicine of Cornell University, Department of Medicine, Pediatrics, and Microbiology and Immunology, New York, United States of America; 3 National and Kapodistrian University of Athens Faculty of Medicine, Department of Pathophysiology, Athens, Greece

**Keywords:** Invasive fungal infections, Antifungal resistance, Hematological malignancies, New antifungal agents

## Abstract

Invasive fungal infections caused by drug-resistant organisms are an emerging threat to heavily immunosuppressed patients with hematological malignancies. Modern early antifungal treatment strategies, such as prophylaxis and empirical and preemptive therapy, result in long-term exposure to antifungal agents, which is a major driving force for the development of resistance. The extended use of central venous catheters, the nonlinear pharmacokinetics of certain antifungal agents, neutropenia, other forms of intense immunosuppression, and drug toxicities are other contributing factors. The widespread use of agricultural and industrial fungicides with similar chemical structures and mechanisms of action has resulted in the development of environmental reservoirs for some drug-resistant fungi, especially azole-resistant Aspergillus species, which have been reported from four continents. The majority of resistant strains have the mutation TR34/L98H, a finding suggesting that the source of resistance is the environment. The global emergence of new fungal pathogens with inherent resistance, such as Candida auris, is a new public health threat. The most common mechanism of antifungal drug resistance is the induction of efflux pumps, which decrease intracellular drug concentrations. Overexpression, depletion, and alteration of the drug target are other mechanisms of resistance. Mutations in the ERG11 gene alter the protein structure of C-demethylase, reducing the efficacy of antifungal triazoles. Candida species become echinocandin-resistant by mutations in FKS genes. A shift in the epidemiology of Candida towards resistant non-albicans Candida spp. has emerged among patients with hematological malignancies. There is no definite association between antifungal resistance, as defined by elevated minimum inhibitory concentrations, and clinical outcomes in this population. Detection of genes or mutations conferring resistance with the use of molecular methods may offer better predictive values in certain cases. Treatment options for resistant fungal infections are limited and new drugs with novel mechanisms of actions are needed. Prevention of resistance through antifungal stewardship programs is of paramount importance.

## Abstract (tr)

İlaca dirençli organizmaların neden olduğu invaziv mantar enfeksiyonları, ağır immün baskılanma altındaki hematolojik kanserli hastalar için bir tehdittir. Profilaktik, ampirik ve önleyici tedaviler gibi güncel anti-fungal tedavi yaklaşımları, direnç gelişiminde büyük bir itici güç olan anti-fungal ajanlara uzun süreli maruz kalma ile sonuçlanmaktadır. Santral venöz kateterlerin uzun süreli kullanımı, bazı anti-fungal ajanların doğrusal olmayan farmakokinetiği, nötropeni, yoğun immün baskılamanın farklı formları ve ilaç toksisitesi direnç gelişimine katkıda bulunan diğer faktörlerdir. Benzer kimyasal yapılara ve etki mekanizmalarına sahip, tarımsal ve endüstriyel fungisitlerin yaygın kullanımı, dört kıtadan bildirilen bazı ilaca dirençli mantarlar, özellikle azole dayanıklı Aspergillus türleri için çevresel kaynakların gelişmesine neden olmaktadır. Dirençli suşların çoğunda bulunan TR34 / L98H mutasyonu, direncin çevresel kaynaklı olduğunu düşündürmektedir. Candida auris gibi doğal dirençli yeni fungal patojenlerin ortaya çıkması, yeni bir halk sağlığı tehdididir. Anti-fungal ilaç direncinin en yaygın mekanizması, hücre içi ilaç konsantrasyonlarını azaltan hücre dışına atım pompalarının uyarılmasıdır. Diğer direnç mekanizmaları arasında ilaç hedefinin aşırı ekspresyonu, tükenmesi ya da değişmesi bulunmaktadır. ERG11 genindeki mutasyonlar, antif-fungal triazollerin etkinliğini azaltarak C-demetilazın protein yapısını değiştirir. Candida türleri, FKS genlerindeki mutasyonlarla ekinokandine dirençli hale gelir. Candida epidemiyolojisinde dirençli albicans-dışı Candida spp. hematolojik kanseri olan hastalarda ön plana çıkmaktadır. Bu popülasyondaki hasta grubunda artmış minimum inhibitör konsantrasyonlarla tanımlanan anti-fungal direnç ile klinik sonuçlar arasında kesin bir ilişki yoktur. Moleküler yöntemlerin kullanımı ile dirence neden olan genlerin veya mutasyonların saptanması, bazı olgularda daha iyi klinik ön görü sağlayabilir. Dirençli fungal enfeksiyonlara yönelik tedavi seçenekleri sınırlıdır ve yeni mekanizmalara sahip ilaçlara ihtiyaç duyulmaktadır. Anti-fungal idame programlarıyla direncin önlenmesi büyük önem taşır.

## Introduction

Invasive fungal infections (IFIs) are associated with increased morbidity and unacceptably high mortality among patients with hematological malignancies (HMs) [1,2]. However, treatment options are limited, including only four chemical classes: polyenes, triazoles, echinocandins, and flucytosine. The expansion of the use of antifungal agents over the last two decades not unexpectedly contributed to the development of antifungal resistance [3,4,5]. Another factor driving the emergence of resistance is the widespread use of agricultural and industrial fungicides with chemical structures and mechanisms of action similar to those of human antifungal agents, resulting in the development of environmental reservoirs for some drug-resistant fungi, especially triazole-resistant *Aspergillus* species [6,7]. Recently, researchers showed that even the household environment may serve as a potential source of triazole-resistant invasive aspergillosis [8].

Antifungal resistance can be either intrinsic or acquired (Table 1) [9,10,11]. Intrinsic drug resistance can occur naturally among certain fungi without previous exposure to antifungal agents, such as fluconazole-resistant *Candida krusei* [9,12]. The emergence of new fungal species with intrinsic resistance to some or all antifungal agents is a new threat. The recent outbreaks of multidrug-resistant *Candida auris *[13] in many hematology centers around the world and the increasing reports of infections caused by panresistant *Lomentospora prolificans* [14,15] are characteristic examples.

Acquired or iatrogenic antifungal resistance is favored by specific risk factors in patients with HMs. Modern early treatment strategies, such as prophylaxis and empirical and preemptive therapy, result in long-term exposure to antifungal agents, which is a major driving force for the development of resistance [5]. Repeated cycles of chemotherapy and/or hematopoietic stem cell transplantation (HSCT) prolong even more the exposure to antifungal agents. Chemotherapy-induced neutropenia limits the pharmacodynamic response to antifungal agents and dictates prolonged therapeutic courses. Indwelling catheters, especially central venous catheters (CVCs), are a major factor for the development of resistance, as their surfaces are often infected by pathogenic fungi and the ensuing biofilm formation does not allow drug penetration, thus rendering the infection refractory to treatment [16,17,18,19]. Nonlinear pharmacokinetics of certain antifungal agents, especially certain triazoles, may result in suboptimal antifungal drug levels, favoring the development of resistance [20,21]. Intraabdominal fungal infections in patients with HMs, such as intraabdominal abscesses, can promote drug resistance because antifungal drug delivery in the abdomen is poor and fungi are exposed to possibly subtherapeutic drug concentrations [22].  

The emergence of antifungal drug resistance has tremendous clinical implications, as it further restricts the already limited antifungal armamentarium, raising concerns among clinicians that we are close to the “post-antifungal” era, in parallel to the “post-antibiotic” era [4,10]. The outlook is similarly grim, as there is a paucity of new antifungal agents with novel mechanisms of action in development [23]. 

The focus of this review will be the emergence of fungal infections with innate or acquired resistance to antifungal agents among patients with HMs. We will visit the many different facets of this complex area, including mechanisms of resistance, epidemiology, clinical implications, and current treatment options. Finally, we will review new antifungal agents in development and the priorities for future research in the field.

## Antifungal-Resistant Invasive Aspergillosis

### Mechanisms of Resistance

Triazole-Resistant *Aspergillus* spp.: Triazoles with activity against *Aspergillus* spp. (i.e. itraconazole, voriconazole, posaconazole, and isavuconazole) are recommended for the treatment of invasive aspergillosis among patients with HMs. Antifungal triazoles act by inhibiting the cytochrome P450 enzyme sterol 14a-demethylase, which converts lanosterol to ergosterol, and is encoded by the gene *CYP51* in filamentous fungi. Inhibition of 14a-demethylase by an azole results in the interruption of biosynthesis of ergosterol, which is fungicidal for molds, as it leads to intracellular accumulation of toxic 14a-methyl sterols and to alterations in cell membrane structure, impairing its permeability and stability and thus the viability of the fungus. Mutations in the *CYP51A* fungal gene alter the structure of the 14a-demethylase, leading to reduced azole binding and thus generating triazole-resistant phenotypes [24,25]. The two most common alterations in *CYP51A* offering resistance to triazoles are tandem repeats in the promoter region of the gene along with gene mutations and point mutations [5]. There are also other non-*CYP51* mechanisms associated with azole resistance [24].

The most frequently identified mechanism of triazole resistance in *Aspergillus fumigatus *involves a 34-bp tandem repeat (TR_34_) in the promoter region of the *CYP51A* gene combined with a substitution of leucine 98 to histidine (TR_34_/L98H). These alterations cause overexpression of the gene [25,26]. Another mechanism of resistance involves a 46-bp tandem repeat in the *CYP51A* promoter region combined with two substitutions: tyrosine 121 for phenylalanine and threonine 289 for alanine (TR_46_/Y121F/T289A) [27]. This modification of the *CYP51A* gene makes *Aspergillus fumigatus* resistant to voriconazole [28]. Finally, a 53-bp tandem repeat in the promoter region of the *CYP51A* gene without any other substitution conferring azole resistance has been detected in environmental [29] and clinical triazole-resistant *Aspergillus fumigatus *strains [30].

Another mechanism of triazole resistance for* Aspergillus s*pp.is nonsynonymous hot-spot mutations in the *CYP51A* gene. Numerous amino acid substitutions associated with reduced susceptibility for triazoles have been reported [24,31,32,33,34,35,36,37,38,39,40]. Recently, many azole-resistant *Aspergillus* isolates were found not to have point mutations in *CYP51A* or promoter duplications, suggesting that alternative mechanisms for azole resistance exist [40,41]. Researchers reported that 43% of 64 azole-resistant *Aspergillus* isolates did not carry a *CYP51A* mutation, indicating that other mechanisms must be responsible [42]. Potential mechanisms conferring resistance include activation of efflux pumps [43]; overexpression of transporter genes [44]; loss of the *algA* gene [45]; the point mutation P88L in *HapE*, an important transcription factor [46]; biofilm formation [43,47]; and cholesterol import by *Aspergillus fumigatus* to overcomeergosterol deprivation [48].

Cryptic *Aspergillus* spp. may be resistant to voriconazole. For example, *Aspergillus calidoustus *typically has elevated minimum inhibitory concentrations (MICs) for voriconazole that exceed CLSI and EUCAST interpretive breakpoints. *Aspergillus lentulus*, which may phenotypically resemble a slowly growing *Aspergillus* *fumigatus*, may also have elevated MICs for voriconazole [24].

Polyene-Resistant *Aspergillus* spp.:Polyeneantifungal agents bind to ergosterol on the cell membrane of the fungus and cause formation of intramembrane channels that kill the cell. Amphotericin B is a first-line treatment for invasive aspergillosis in patients with HMs. Although it has been used since 1957, emergence of resistance is usually not an issue and typically involves selection of inherently resistant strains. Development of acquired resistance during therapy is rare [5]. The most common amphotericin B-resistant species include *Aspergillus terreus, Aspergillus flavus, Aspergillus nidulans, Aspergillus calidoustus, *and* Aspergillus lentulus *[49,50,51]*.* The main mechanism of resistance is believed to be the modification of the cell membrane, by diminishing its ergosterol content [51].

Researchers have found that previous treatment with triazoles also may reduce the amount of membrane ergosterol in *Candida* spp. resistant to amphotericin B [52]. Reduction of membrane ergosterol renders* Cryptococcus neoformans *less susceptible to amphotericin B [53]. Whether this mechanism also confers polyene resistance to *Aspergillus* spp. is uncertain.

## Epidemiology

Triazole-resistant *Aspergillus fumigatus* has been described in the Netherlands since 1999, with an estimated prevalence of 6.0%-12.8% of patients with invasive aspergillosis [6]. In 2007, infections caused by triazole-resistant *Aspergillus fumigatus* were reported in hematology patients from six different hospitals in the Netherlands [25]. One year later, another Dutch hospital noted that 28.1% of 32 patients with invasive aspergillosis had an azole-resistant isolate of *Aspergillus fumigatus* [54]. The predominant mechanism of resistance of clinical isolates from patients in different hospitals was TR34/L98H, a finding suggesting that the source of resistance was the environment [54,55]. Subsequent studies from the Netherlands [55] and the United Kingdom [56] showed that, from 1994 to 2009, the incidence of triazole-resistant aspergillosis rapidly increased to 20%. Recently, a prospective study on the prevalence and the mechanisms of azole-resistance was conducted among 22 centers in 19 European countries [25]. Triazole-resistant *Aspergillus fumigatus* isolates have been reported in 11 countries, although the prevalence ranged widely, from 0% to 26%, among the participating centers and even among centers from the same country. The overall triazole resistance prevalence was 3.2% [25]. To date, triazole-resistant clinical isolates of *Aspergillus fumigatus* have been reported in the majority of European counties [24], as well as Turkey [56].

Most reports of triazole-resistant *Aspergillus* spp. have originated from Europe, but recently researchers from four continents reported increasing numbers of infections caused by resistant *Aspergillus* strains [34,57,58,59,60,61], suggesting that azole resistance is a global threat.

## Clinical Significance

Data on the clinical significance of triazole resistance are limited and contradictory. In vitro studies have shown that the presence of triazole resistance mechanisms is associated with reduced susceptibility of *Aspergillus fumigatus* to all azoles [62], including the recently licensed isavuconazole [63,64,65]. Several studies have shown that triazole resistance is associated with treatment failure, especially among patients with HMs [24,28,29,36,39]. In a study from India, invasive aspergillosis caused by a resistant isolate was associated with a significantly higher mortality rate (88%) compared with that of aspergillosis caused by wild-type isolates (30%-50%) [66]. On the contrary, in a retrospective study from the United States, higher azole MICs were not correlated with outcome of aspergillosis in patients with HMs or HSCT recipients [67]. Clearly, more data are needed to delineate the clinical significance of triazole resistance in *Aspergillus* spp.

## Treatment

Due to the low worldwide prevalence of azole-resistant aspergillosis, there are no clinical studies on its treatment. In 2015, an expert panel published an opinion paper on how to treat azole-resistant aspergillosis [68]. They suggested that in areas with high (>10%) environmental resistance, first-line therapy should be liposomal amphotericin B or a combination of voriconazole and an echinocandin. These suggestions require meticulous surveillance studies to define areas of high resistance; such studies are not always feasible.

## Antifungal-Resistant Invasive Candidiasis

### Mechanisms of Resistance

Triazole-Resistant *Candida* spp.: Antifungal azoles act by inhibiting the enzyme sterol 14a-demethylase, resulting in the interruption of biosynthesis of ergosterol, which is an essential *Candida* cell membrane component. The inhibition of ergosterol synthesis may be fungicidal for molds, but only fungistatic for yeasts. Several mechanisms confer azole resistance to *Candida* spp. [18]. The most common mechanism is the induction of efflux pumps, which decrease the intracellular drug concentration. Efflux pumps are encoded by various genes belonging to the ATP-binding cassette superfamily or to the major facilitator superfamily [69]. The transcription of these genes is regulated by transcription factors, such as *Tac1* and *Mrr1* for *Candida albicans *and *CgPdr1 *for *Candida glabrata* [69]. Overexpression or alteration of the drug target, 14a-demethylase, is another mechanism of resistance. Numerous point mutations in the *ERG11* gene, usually after exposure to fluconazole, can generate structural changes in the active site of the demethylase, causing reduced target affinity and thus triazole resistance [70]. Overexpression of *ERG11* [71] and loss of function of the sterol Δ5,6-desaturase gene (*ERG3*) [72] also confer azole resistance. Loss of function of the sterol Δ5,6-desaturase gene in* Candida glabrata* may also result in resistance to amphotericin B. These mechanisms can occur either alone or concurrently in a single isolate and may lead to cross-resistance to many azoles.

Echinocandin-Resistant *Candida* spp.: The mechanism of action of the echinocandins is inhibition of (1,3)-b-D-glucan synthesis [73]. Beta-D-glucans are cross-linked to chitin and mannoproteins, providing structural integrity to cell walls of various fungi. Echinocandins are fungicidal for *Candida *spp., as b-D-glucan accounts for approximately 30%-60% of the cell wall mass in *Candida* species [73]. Conversely, among filamentous fungi, echinocandins have only fungistatic effects, as the cell wall contains less glucan, concentrated at the apical tips and branching points of hyphae.

Echinocandins exert their antifungal activity by binding to the enzyme FKS, which catalyzes the synthesis of (1,3)-b-D-glucans. Glucan synthase has two catalytic subunits, FKS1 and FKS2, encoded by their respective *FKS* genes. *Candida* species become echinocandin-resistant by genetic acquisition of mutations in *FKS* genes, which encode amino acid substitutions in two narrow hot-spot regions of *FKS1* for all *Candida* species and *FKS2 *for *C. glabrata* [74]. The most common (>90%) *FKS1* substitutions among echinocandin-resistant *Candida albicans *isolates occur at Ser-641 or Ser-645 [74]. In* Candida glabrata*, the most common amino acid substitutions occur in *FKS2* [75].

Resistance to two or more classes of antifungal agents further augments the threat of *Candida* *glabrata* in patients with HMs. *Candida glabrata *bloodstream isolates from patients with HMs developed cross-resistance to both triazoles and echinocandins [76]. While the molecular events leading to triazole and echinocandin resistance may occur independently, one potential unifying mechanism is the development of DNA mismatch-repair gene mutations, which lead to “hypermutable” clinical strains [12].

Polyene-Resistant *Candida* spp.: *Candida* species with acquired resistance to polyenes are uncommon, although researchers have reported cases of *Candida albicans, Candida krusei, Candida glabrata, Candida tropicalis, Candida rugosa, Candida lusitaniae*, and* Candida guilliermondii* with high MICs to amphotericin B [5,18]. The main mechanism of resistance involves a reduction in cell membrane ergosterol, which is the biological target of amphotericin B. Reduction of ergosterol can be caused by previous treatment with triazoles, which lowers membrane sterol concentrations, or mutations affecting sterol biosynthesis, such as defects in *ERG1*, *ERG2*, *ERG3*, *ERG4*, *ERG6*, and *ERG11* [18,77].

Biofilm Formation and *Candida* Resistance: Biofilm formation on artificial devices, especially CVCs, is an essential factor driving the development of drug-resistant *Candida* spp. in patients with HMs. Antifungal drugs do not achieve therapeutic levels within the biofilm because they are trapped in a glucan-rich matrix polymer. The hypoxic environment within biofilms results in a metabolic stress response that leads to increased MICs to triazoles. Moreover, once the *Candida* strain is embedded in the biofilm, it may not need to be resistant in order to grow despite adequate antifungal treatment and may cause breakthrough candidemia [19].

### Epidemiology

Antifungal drug resistance has emerged through the development of acquired resistance and an epidemiological shift in the distribution of *Candida *species towards inherently less susceptible non-*albicans* species [16]. In large-scale surveillance studies of bloodstream isolates, the overall prevalence of *Candida albicans* resistance is less than 1% [78]. Resistance rates are higher among non-*albicans* *Candida* species, notably *Candida glabrata*, reaching 2%-4% in most epidemiological prevalence studies [79]. A trend towards increasing rates of *Candida glabrata* resistance has been noted, as the proportion of nonsusceptible isolates increased from 4.2% in 2008 to 7.8% in 2014 [80], while some institutions reported resistance rates close to 10% [75]. In hematology patients, a rise in *Candida glabrata *with echinocandin and azole resistance and cross-resistance to two or more antifungal classes (multidrug resistance) has been reported, mainly in the United States, but not in Europe [81]. In a European study of candidemia among hematology patients, in vitro resistance to at least one antifungal agent was observed for 27% of *Candida* isolates [17].

The problem of antifungal-resistant yeast infections has been aggravated by recent epidemiological changes. A shift in the distribution of candidemia-associated *Candida *species towards more resistant non-*albicans *species, such as *Candida parapsilosis*,* Candida tropicalis*,* Candida glabrata*, and *Candida krusei*, has been reported among patients with HMs in both the United States and Europe [16,17]. In addition, the recent emergence of *Candida auris,* an uncommon species that exhibits both multidrug resistance and strong potential for nosocomial transmission, raises concerns worldwide [82]. Cases and hospital outbreaks of *Candida auris* invasive infections have been reported from four continents, mainly among patients with HMs, with high mortality [82,83].

### Clinical Significance

There are no clinical studies showing a definite association between in vitro susceptibility testing and outcomes of invasive candidiasis in neutropenic patients [4,18,19], with the exception of *Candida glabrata*, where clinical studies demonstrated that infection with an echinocandin-resistant strain was associated with worse outcomes [9,75]. Clinical failure was associated with the presence of the *FKS* mutation and not MIC values [9]. Finally, the recent epidemiological shift of *Candida* species distribution towards non-*albicans* species in patients with HMs [16] has an impact on outcomes as many non-*albicans* species, especially *Candida glabrata* and *Candida krusei*, exhibit higher resistance rates and higher mortality [16,17].

### Treatment

There are no clinical studies on the optimal initial treatment of patients with or at risk for antifungal-resistant invasive *Candida* infections. Current guidelines for treatment of candidiasis recommend lipid formulation of amphotericin B (3-5 mg/kg daily) for patients with suspected azole- and echinocandin-resistant *Candida* infections [84]. This recommendation is characterized as “strong” but is based on “low-quality evidence”. Regarding the emerging problem of multidrug-resistant *Candida glabrata* infection, there are no good clinical data on the optimal treatment. The best strategy for the initial treatment of suspected or documented resistant *Candida* infection is to be tailored according to individual risk factors and the local epidemiology [18].

### Antifungal Resistance in Fungal Infections Caused by Rare Molds and Non-Candida, Non-Cryptococcus Yeasts

The frequency of invasive fungal disease caused by resistant filamentous fungi other than *Aspergillus* is increasing. The majority of these rare molds are Mucorales, hyalohyphomycetes (*Fusarium *spp.,* Scedosporium *spp.), and dematiaceous fungi and they occur mainly in heavily immunosuppressed patients with HMs [85]. The TRANSNET study reported that among 983 IFIs identified in 875 HSCT recipients, 8% were mucormycosis and 14% of infections were caused by other filamentous fungi [86]. The intrinsic resistance of many of these rare fungi to antifungal agents is of concern. Mucorales species are resistant to some triazoles, while multidrug resistance has been reported for *Fusarium *spp.,* Scedosporium *spp., and dematiaceous fungi.

Although *Candida* infections comprise the vast majority of yeasts growing in blood cultures, clinicians should be aware that a substantial proportion of fungemia cases are caused by non-*Candida*, non-*Cryptococcus* yeasts [87], such as *Trichosporon asahii*, *Magnusiomyces (Blastoschizomyces) capitatus, Saccharomyces cerevisiae, Malassezia *spp.,* Saprochaete (Geotrichum*)spp., and *Rhodotorula* spp. The majority of these rare yeasts are intrinsically resistant to one or more classes of antifungal agents, and infections occur frequently as breakthrough infections in hematology patients receiving antifungals and with a CVC in place [87,88]. For instance, *Trichosporon* spp. are resistant to echinocandins and to the fungicidal activity of polyenes, while *Rhodotorula* spp. are resistant to the triazoles [18]. In vitro susceptibility testing is not always useful in patients with infections caused by less frequent opportunistic yeast or mold infections. In these patients, breakpoints are not based on data derived from clinical responses or outcomes but only from epidemiological cut-off values and pharmacokinetic and pharmacodynamic data from animal models [89].

### Diagnostic Tests for the Detection of Fungal Resistance

Isolation of the infecting fungus through conventional culture of biological fluids and tissues, identification to the species level, and in vitro testing to determine the susceptibility to antifungal agents is the current standard for the diagnosis of IFIs caused by resistant fungi and for decision making [90]. Species identification is time-consuming, prompting physicians to initiate empirical treatment until the results become available. Newer methods, including MALDI-TOF mass spectroscopy and T2 magnetic resonance assay, allow rapid species identification with excellent sensitivity and specificity [90,91]. Antifungal susceptibility testing is recommended for the triazoles against all bloodstream *Candida* isolates and for the echinocandins against resistant species, such as *Candida glabrata* and *Candida parapsilosis* isolates [84]. As mentioned previously, clinical breakpoints are only available for certain species of fungi and are not useful for the diagnosis of resistance, as they do not always correlate with clinical outcomes, especially in patients with HMs [18,19,90]. Thus, a low MIC value does not necessarily predict successful treatment and an elevated MIC does not automatically predict treatment failure.

Currently, only polymerase chain reaction (PCR) has the potential for early detection of resistance [92]. Even PCR, though, has its drawbacks, such as low sensitivity for detection of resistance markers and difficulty in differentiating colonization from invasive infection or a living from a dead organism [93]. Therefore, clinicians should be cautious as to how to interpret these non-culture-based diagnostic tests in everyday clinical practice and for decision making. New molecular detection methods, including HRMA/PCR, microarrays, and metagenomic shotgun sequencing, are under development and hold promise for the future [92].

### New Antifungal Agents for Resistant Fungi

IFIs caused by drug-resistant organisms are an emerging threat to heavily immunosuppressed patients with HMs. Therefore, there is an urgent need for new antifungals with activity against resistant fungi. It should be underlined, though, that fungi are eukaryotes, just like human cells; thus, discovering new antifungal agents not interfering with human cells is challenging.

Recent developments in fungal functional genomics, proteomics, and gene mapping allowed the discovery of potential new drug targets that could offer additional options to treat resistant fungal infections [94]. Cellular and biochemical targets of investigational agents against drug-resistant fungal pathogens include metabolic pathways (such as the glyoxylate cycle, iron metabolism, and heme biosynthesis), cell wall and cell membrane components, signal transduction pathways (such as MAP kinase), and gene expression. However, there is a paucity of novel antifungal compounds in preclinical or clinical development, as the majority of these new antifungal agents are in the very early stages of development.

SCY-078 is the first orally bioavailable inhibitor of (1,3)-b-D-glucan synthesis of the fungal cell wall. A triterpene derivative, SCY-078 has demonstrated in vitro and in vivo activity against all tested *Candida* spp., including* Candida auris*, as well as triazole-resistant and echinocandin-resistant *Candida* spp. [94]. Its spectrum includes *Aspergillus* spp., where it may be particularly effective in combination with anti-mold triazoles. E1210 is a novel isoxazolyl-bis-pyridine wall-active antifungal compound that inhibits inositol acylation of mannosylated cell wall proteins, resulting in arrest of fungal growth [94]. The antifungal spectrum includes most yeast with the exception of *Candida krusei* and molds, including isolates resistant to triazoles and polyenes. Biafungin (CD101) is a novel, long-acting, semisynthetic echinocandin derivative of anidulafungin that is currently in phase III clinical studies. In vitro susceptibility testing showed that biafungin has activity against caspofungin-resistant *Candida* strains containing *FKS* mutations [95]. Other antifungal agents under development include F901318 (dihydroorotate dehydrogenase inhibitor), VT-1598 (metalloenzyme inhibitors of CYP51), and ASP2397 (hydroxamate siderophores-like agent) [94].

### Future Research Directions in Fungal Resistance

Invasive infections caused by resistant fungi are emerging global problems of public health, associated with increased morbidity and mortality, particularly among patients with HMs. There are unanswered questions and unmet needs in all areas of knowledge of fungal resistance, including epidemiology, diagnostics, therapeutics, prevention, and education, that require expertise from many different disciplines to be addressed [96].

The emerging epidemiological data raise intriguing questions: why is the prevalence of azole resistance in *Aspergillus* so variable? The frequency of resistance may vary considerably, not only between continents and countries but also between hospitals within the same country, between departments, or between risk groups within the same hospital [97,98,99]. Is this under- or overreporting, suboptimal sampling, and/or technical issues in *Aspergillus fumigatus *isolation and resistance detection? Alternatively, are there any geoclimatic factors that create ecological niches favoring the spread of resistance? Obviously, general surveillance studies are not sufficient to capture the problem. In the future, meticulous well-funded epidemiological studies targeted to specific high-risk groups, especially patients with HMs, are necessary.

Development and implementation of laboratory diagnostic tools should be a priority for future research in the field of resistant fungal infections, as current technology does not allow rapid species identification and assessment of resistance. Development of interpretive breakpoints for fungal infections in neutropenic patients with HMs is an unmet need. New molecular technologies for the prompt and accurate detection of genes and mutations associated with fungal resistance are urgently needed.

The existing antifungal agents are not sufficient to confront the growing trend of resistance. The limited antifungal armamentarium should be enriched with agents with novel mechanisms of action to overcome resistance. A fascinating direction for future research is the development of new antifungal agents that do not kill or inhibit the growth of fungi but impair key virulence properties, such as invasion or adherence.

Prevention of fungal resistance should be at the core of future research. Antifungal stewardship programs should ensure that there is an indication for antifungal therapy, that the appropriate antifungal agent is selected, and that the dosage, route of administration, and duration are optimal and that de-escalation is implemented when feasible. A robust antifungal stewardship program might have beneficial effects on the prevention of resistance. Understanding the pathophysiology of biofilm formation and reducing the use of CVCs might also prevent the development of catheter-related resistant fungal infections.

## Figures and Tables

**Table 1:**
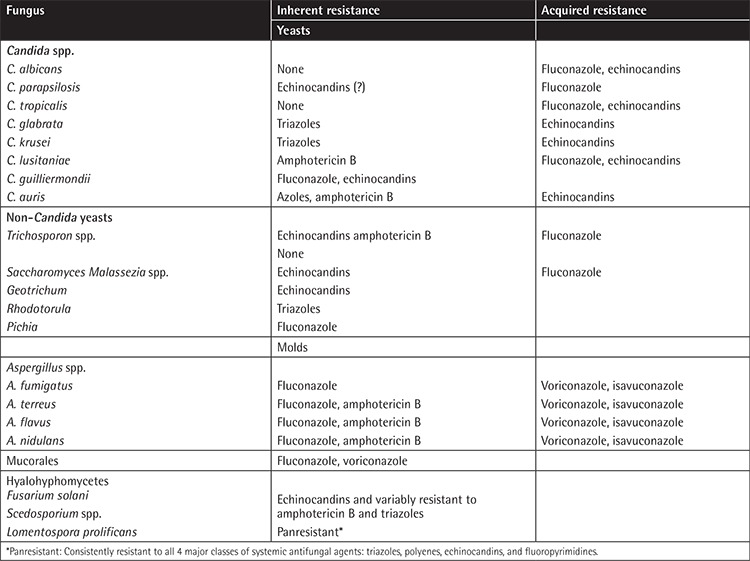
Inherited and acquired resistance reported among pathogenic fungi infecting patients with hematological malignancies.
